# Long-Term Clinical Practice Experience with Cinacalcet for Treatment of Hypercalcemic Hyperparathyroidism after Kidney Transplantation

**DOI:** 10.1155/2015/292654

**Published:** 2015-03-10

**Authors:** Ursula Thiem, Alois Gessl, Kyra Borchhardt

**Affiliations:** ^1^Division of Nephrology and Dialysis, Department of Medicine III, Medical University of Vienna, Spitalgasse 23, 1090 Vienna, Austria; ^2^Division of Endocrinology and Metabolism, Department of Medicine III, Medical University of Vienna, Spitalgasse 23, 1090 Vienna, Austria; ^3^Dialysis Institute Klagenfurt, Heiligengeistplatz 4/III, 9020 Klagenfurt, Austria

## Abstract

Within this prospective, open-label, self-controlled study, we evaluated the long-term effects of the calcimimetic cinacalcet on calcium and phosphate homeostasis in 44 kidney transplant recipients (KTRs) with hypercalcemic hyperparathyroidism by comparing biochemical parameters of mineral metabolism between pre- and posttreatment periods. Results are described as mean differences (95% CIs) between pre- and posttreatment medians that summarize all repeated measurements of a parameter of interest between the date of initial hypercalcemia and cinacalcet initiation (median of 1.6 (IQR: 0.6–3.8) years) and up to four years after treatment start, respectively. Cinacalcet was initiated after 1.8 (0.8–4.7) years posttransplant and maintained for 6.2 (3.9–7.6) years. It significantly decreased total serum calcium (−0.30 (−0.34 to −0.26) mmol/L, *P* < 0.001) and parathyroid hormone levels (−79 (−103 to −55) pg/mL, *P* < 0.001). Serum levels of inorganic phosphate (Pi) and renal tubular reabsorption of phosphate to glomerular filtration rate (TmP/GFR) increased simultaneously (Pi: 0.19 (0.15–0.23) mmol/L, *P* < 0.001, TmP/GFR: 0.20 (0.16–0.23) mmol/L, *P* < 0.001). In summary, cinacalcet effectively controlled hypercalcemic hyperparathyroidism in KTRs in the long-term and increased low Pi levels without causing hyperphosphatemia, pointing towards a novel indication for the use of cinacalcet in KTRs.

## 1. Introduction

Persistent hyperparathyroidism after kidney transplantation is frequently associated with hypercalcemia [1] and is considered a risk factor for progressive bone loss [2] and fractures [3] and vascular calcification [4], as well as the development of tubulointerstitial calcifications of renal allografts and graft dysfunction [5]. In a subgroup of patients, hypophosphatemia occurs in addition to hypercalcemia [6] and poses another risk factor for posttransplant bone disease [7]. Consequently, correcting disturbances in mineral homeostasis is regarded as an important goal in the care of kidney transplant patients.

In the management of severe posttransplant hypercalcemic hyperparathyroidism, parathyroidectomy is regarded as the gold-standard [8]. Therapeutic options are limited for patients with milder forms of hypercalcemic hyperparathyroidism and those not eligible for parathyroid surgery. Additionally, treatment of hypophosphatemia is challenging in hypercalcemic patients as the use of phosphate supplements involves the risk of developing soft tissue calcifications [9–11].

The calcimimetic cinacalcet offers a new treatment strategy for these patients. While cinacalcet has been successfully used to control secondary hyperparathyroidism in chronic hemodialysis patients [12, 13] and was shown to reduce the risk of parathyroidectomy in these patients [14], drug approval for the use in kidney transplant recipients is currently pending (clinicaltrials.gov NCT00975000). A recent meta-analysis of 21 nonrandomized studies including a total of 411 kidney transplant recipients with hypercalcemic hyperparathyroidism showed that cinacalcet corrects hypercalcemia and lowers parathyroid hormone (PTH) levels also in kidney transplant patients [15]. The majority of these studies, however, had a relatively short follow-up period of one year or less. Due to the slow regression of hyperparathyroidism after transplantation [16], treatment with cinacalcet may be required for a prolonged period in these patients. So far, only few data on the long-term use of cinacalcet in kidney transplant recipients are available [17–19].

Herein, we report our long-term clinical experience with cinacalcet in 44 kidney transplant recipients with hypercalcemic hyperparathyroidism gathered within a prospective, open-label, self-controlled study. As the majority of the participants had long-standing hypercalcemic hyperparathyroidism following transplantation (up to 11 years), we assessed the effects of cinacalcet on mineral and bone metabolism by comparing pre- and posttreatment periods.

## 2. Materials and Methods

### 2.1. Study Design and Setting

This prospective, open-label, self-controlled study is a long-term follow-up to a previously reported study that examined the effect of six-week cinacalcet treatment on calcium homeostasis after kidney transplantation [20]. It was approved by the Ethics Committee of the Medical University of Vienna (Institutional Review Board Approval Number 211/2005) and registered in the European Clinical Trials Register (EudraCT 2005-004148-29). Informed written consent was obtained from all participants.

Between August 2005 and October 2008, 44 stable kidney transplant recipients with hypercalcemic hyperparathyroidism were included in the study. Patients were recruited from the nephrology outpatient unit at the Medical University of Vienna, Austria. All of the patients were treated with cinacalcet, and biochemical parameters of mineral and bone metabolism were prospectively assessed over time. The patients were followed until death, return to dialysis, or December 31, 2013, regardless of whether treatment with cinacalcet was still ongoing.

Moreover, for each patient, we identified the time point when total serum calcium levels initially exceeded 2.60 mmol/L following transplantation and persisted above this threshold thereafter. Starting from the date of initial hypercalcemia, we retrospectively analyzed the course of biochemical parameters of interest over time until initiation of cinacalcet. Thereby, each patient served as his or her own control.

### 2.2. Patients

Hypercalcemic hyperparathyroidism was defined as total serum calcium above 2.60 mmol/L and intact PTH above 60 pg/mL and had been ascertained at three prestudy screening visits before the patients were included in the study. The median timeframe of the screening period before inclusion was 2.2 (interquartile range (IQR): 1.4 to 3.5) months. Patients with hypercalcemia that was possibly related to treatment with active vitamin D analogues were included only if hypercalcemia persisted after withdrawal of active vitamin D analogues. Patients with a history of parathyroidectomy were excluded. Nevertheless, we included one patient who had undergone subtotal parathyroidectomy and had presented with an inoperable hyperplastic parathyroid gland that adhered to the Vena cava. This patient continued having elevated total serum calcium levels between 2.70 and 2.80 mmol/L and intact PTH levels around 100 pg/mL (estimated glomerular filtration rate (eGFR) around 50 mL/min/1.73 m^2^) for one year after parathyroid surgery. Thus, we treated this patient the same way as non-parathyroidectomized patients.

### 2.3. Treatment with Cinacalcet

Cinacalcet (Mimpara, Sensipar, Amgen Inc., Thousand Oaks, USA) was initiated at 30 mg once daily with dinner. Depending on the individual tolerability, the dose of cinacalcet was titrated to achieve total serum calcium levels within the normal range. The maximum daily dose was 180 mg, administered as 90 mg twice daily. In case of side effects, cinacalcet at any dose was given twice daily. If intact PTH levels were not controlled with cinacalcet alone, the participants additionally received active vitamin D analogues. The participants did not receive any phosphate supplements or bisphosphonates during treatment with cinacalcet. The use of nutritional vitamin D (cholecalciferol) was allowed at discretion of the physician in charge. Concomitant medication was maintained in each case on an individual basis.

### 2.4. Follow-Up and Data Collection

Data relevant to this study were collected as part of routine clinical practice. By using the information management software of the Vienna General Hospital we extracted relevant laboratory results for each patient, starting from the date of initial hypercalcemia following transplantation until the last visit (including laboratory results obtained during hospitalizations). The number of measurements of the biochemical parameters of interest varied among the patients depending on the individual course of disease and, thus, the frequency of visits at the nephrology outpatient unit and the patient's need for hospitalization. In general, the patients visited the nephrology outpatient unit every week during the first four months after transplantation. Thereafter, the patients were typically scheduled every two to four weeks for the first year after transplantation. Long-term kidney transplant recipients were seen every two to four months. At every visit routine blood samples were obtained (i.e., approximately 12 hours after intake of cinacalcet) including measurements of total serum calcium, serum levels of inorganic phosphate, and serum and urinary creatinine. Urinary calcium and phosphate concentrations were measured on a three-monthly basis in spontaneous urine samples or, if available, in 24-hour-urine samples. Intact PTH, bone specific alkaline phosphatase, osteocalcin, and C-telopeptide levels were measured approximately every three to six months. Moreover, any symptoms possibly related to the intake of cinacalcet were recorded in the patients' charts.

### 2.5. Biochemical Analyses

Routine blood samples obtained at every visit at the nephrology outpatient unit were assayed at the Central Laboratory of the Medical University of Vienna. Serum and urinary calcium, phosphate, and creatinine levels were determined with standard assays. Fractional calcium clearance was calculated as (calcium_urine_ × creatinine_serum_ × 100)/(calcium_serum_ × creatinine_urine_). Renal tubular reabsorption of phosphate to glomerular filtration rate (TmP/GFR) was calculated as phosphate_serum_ − ((phosphate_urine_ × creatinine_serum_)/creatinine_urine_). Estimated GFR was calculated by the abbreviated Modification of Diet in Renal Disease formula as 186 × creatinine_serum_^−1.154^ × age^−0.203^ × 0.742 (if female). Intact PTH was determined by a commercially available electrochemiluminescence immunoassay (Elecsys, Roche, Switzerland). Osteocalcin and bone specific alkaline phosphatase were determined to assess osteoblast activity and C-telopeptide to assess osteoclast activity. Osteocalcin and C-telopeptide were assayed by an electrochemiluminescence immunoassay (Elecsys, Roche, Switzerland). Bone specific alkaline phosphatase was measured by an enzyme-linked immunosorbent assay (Metra Biosystems Behring Diagnostic, Germany) and from 2010 on by a two-site sandwich chemiluminescence immunoassay (Liaison BAP OSTASE, Diasorin, USA). Bone specific alkaline phosphatase values obtained by these two methods were converted as follows: bone specific alkaline phosphatase concentration (ng/mL) = bone specific alkaline phosphatase activity (U/L) × 0.64 + 1.6.

### 2.6. Statistical Analysis

Baseline characteristics are presented as medians and IQRs for continuous variables and counts and frequencies for categorical variables.

To assess the effect of cinacalcet on biochemical parameters of mineral and bone metabolism, we compared pre- and posttreatment periods in each patient using summary statistics. Thereby, each patient served as his or her own control. For each patient, a pretreatment median was calculated that summarizes all repeated measurements of a parameter of interest obtained between the date of initial hypercalcemia and initiation of cinacalcet. By using Student's paired *t*-test, the pretreatment median was compared to the posttreatment median that summarizes all repeated measurements of a parameter of interest up to four years after initiation of cinacalcet. The first three months following initiation of cinacalcet were excluded from the summary median as in some patients the dose of cinacalcet was titrated over several weeks until a steady state was achieved. As three patients had a follow-up of three months or less, only 41 patients were included in the analysis. Moreover, if cinacalcet was discontinued temporarily, the values were excluded from the summary median. In patients who returned to dialysis, laboratory values were excluded from the summary median as soon as the patient reached chronic kidney disease stage 5 (eGFR < 15 mL/min/1.73 m^2^). In this case, serum levels of inorganic phosphate and intact PTH levels strongly increase, posing a potential bias for the analysis. Differences between the pre- and posttreatment periods are presented as mean differences and 95% confidence intervals (CI). Subgroup analysis between short- and long-term transplant recipients was performed using Student's *t*-test.

For graphical illustration of variations in biochemical parameters of interest (Figures 2 to 7) before and after initiation of cinacalcet, we again used summary statistics in order to balance the effect of short-term variations. For each patient, quarterly median values were obtained by summarizing all repeated measurements of a parameter of interest over a three-month period starting from the date of cinacalcet initiation. If cinacalcet was discontinued temporarily, the values were excluded from the summary median. Graph Pad Prism 5 was used for visualization.

Moreover, we used the reverse Kaplan-Meier method [21] to estimate median treatment and observation time, with the event defined as patients alive at the last follow-up, and death and dialysis as censored observations. Kaplan-Meier plots were generated with SPSS Statistics 20.

To study the side effects of cinacalcet, we calculated the exposure-adjusted incidence rate as 100 × (number of patients with side effects/total number of patient-years of exposure). The 95% confidence interval of the exposure-adjusted rate was calculated as exposure-adjusted rate ± (1.96 × (exposure-adjusted rate/√number of patients with side effects)).

## 3. Results

### 3.1. Patients

Forty-four stable kidney transplant recipients with hypercalcemic hyperparathyroidism were included in the study, out of whom forty-one patients (93%) showed an early onset of hypercalcemia within the first six months after transplantation, two patients developed hypercalcemia within the second posttransplant year, and only one patient had late onset of hypercalcemia after 7.5 years.

**Figure 1:**
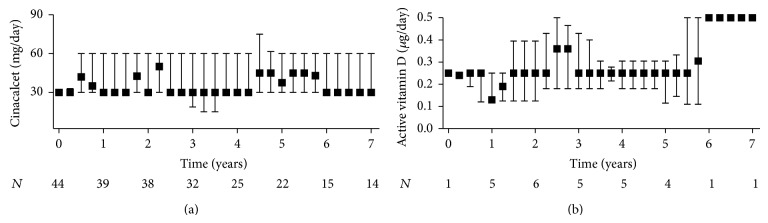
Daily dose of (a) cinacalcet and (b) active vitamin D analogues used during the study. (a) Cinacalcet was initiated in 44 kidney transplant recipients with hypercalcemic hyperparathyroidism and treatment was maintained for a median duration of 6.2 (3.9 to 7.6) years. (b) During the study eight patients received active vitamin D analogues in addition to cinacalcet. The median duration of treatment was 3.3 (2.4 to 4.1) years. Data are presented as medians and interquartile ranges.

Cinacalcet was initiated after a median period of 1.8 (0.8 to 4.7) years after transplantation. Before treatment start, hypercalcemic hyperparathyroidism was ascertained at three screening visits. Total serum calcium and intact PTH levels were 2.76 (2.70 to 2.84) mmol/L and 163 (102 to 246) pg/mL at the first and 2.75 (2.70 to 2.81) mmol/L and 157 (99 to 262) pg/mL at the second screening visit, respectively. In a subgroup of 32 patients technetium 99 sestamibi scans and/or neck ultrasounds were performed showing adenoma as the underlying disease in 15 patients and four-gland hyperplasia in 17 patients as reported previously [20]. Demographic characteristics and laboratory findings at baseline (i.e., the third screening visit) are summarized in Table 1. Cinacalcet was initiated at 30 mg, with the exception of one patient who had had severe hypercalcemia (around 3 mmol/L) for almost half a year and therefore received 60 mg as a starting dose. At some point during the study, eight patients received active vitamin D analogues in addition to cinacalcet. The median doses of cinacalcet and active vitamin D analogues used during the study period are depicted in Figure 1. The median treatment period with cinacalcet was 6.2 (3.9 to 7.6) years as determined by the reverse Kaplan-Meier method. Patients in whom cinacalcet was discontinued were followed to the time of death or return to dialysis or until December 2013. Thus, the median observation period of 7.0 (6.1 to 7.7) years was longer than the treatment period.

During the study period, the immunosuppressive regimen was changed in seven patients, with six having been switched from cyclosporine A to tacrolimus and one patient from sirolimus to tacrolimus. In another patient, azathioprine was discontinued.

During the entire observation period, seven participants returned to dialysis because of graft loss due to chronic transplant failure. In these patients, an eGFR below 15 mL/min/1.73 m^2^ occurred after a median period of 10.6 (5.6 to 11.9) years after transplantation. They had received cinacalcet for a median period of 4.1 (3.2 to 4.6) years, and by the time they reached chronic kidney disease stage 5 cinacalcet had been discontinued in three patients. Moreover, seven participants died during the entire observation period. Causes of death were lung cancer (*n* = 2), hepatocellular carcinoma (*n* = 1), posttransplant lymphoma (*n* = 1), septic shock and multiple organ failure (*n* = 1), and acute respiratory distress syndrome (*n* = 1). The cause of death is unknown in one patient. These patients had received cinacalcet for a median period of 1.8 (0.8 to 4.2) years, and by the time of death cinacalcet had been discontinued in one patient. All cases of death were likely unrelated to the study drug.

### 3.2. Biochemical Parameters of Mineral and Bone Metabolism

**Figure 2:**
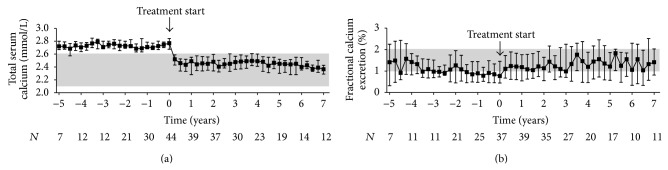
Calcium homeostasis in kidney transplant recipients treated with cinacalcet for hypercalcemic hyperparathyroidism. Variations of (a) total serum calcium levels and (b) urinary fractional calcium excretion before and after initiation of cinacalcet (*t* = 0) are plotted over time. Starting point of the pretreatment period is the date when the patients initially presented with hypercalcemia following transplantation. For both pre- and posttreatment period, quarterly median values were obtained for each patient by summarizing all repeated measurements over a three-month period starting from *t* = 0. Data are presented as medians and interquartile ranges. The shaded area depicts the reference range.

**Figure 3:**
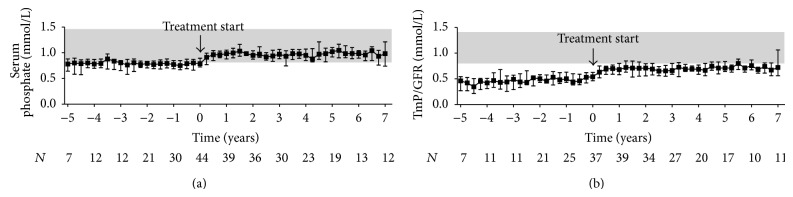
Phosphate homeostasis in kidney transplant recipients treated with cinacalcet for hypercalcemic hyperparathyroidism. Variations of (a) serum levels of inorganic phosphate and (b) renal tubular reabsorption of phosphate to glomerular filtration rate (TmP/GFR) before and after initiation of cinacalcet (*t* = 0) are plotted over time. Starting point of the pretreatment period is the date when the patients initially presented with hypercalcemia following transplantation. For both pre- and posttreatment period, quarterly median values were obtained for each patient by summarizing all repeated measurements over a three-month period starting from *t* = 0. Data are presented as medians and interquartile ranges. The shaded area depicts the reference range.

**Figure 4:**
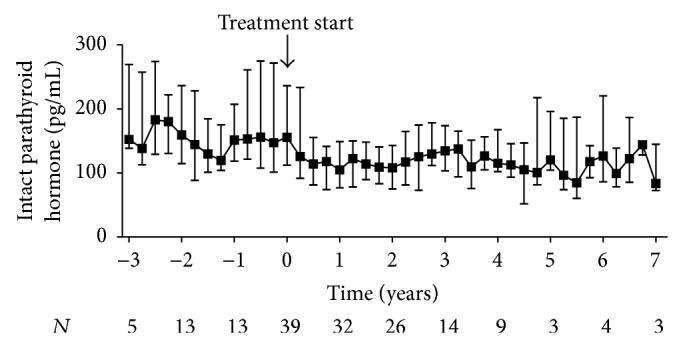
Parathyroid hormone levels in kidney transplant recipients treated with cinacalcet for hypercalcemic hyperparathyroidism. Variations of intact parathyroid hormone levels before and after initiation of cinacalcet (*t* = 0) are plotted over time. Starting point of the pretreatment period is the date when the patients initially presented with hypercalcemia following transplantation. For both pre- and posttreatment period, quarterly median values were obtained for each patient by summarizing all repeated measurements over a three-month period starting from *t* = 0. Values were excluded if a patient received active vitamin D in addition to cinacalcet. Data are presented as medians and interquartile ranges.

Comparison of the patients' pre- and posttreatment median values of total serum calcium revealed that cinacalcet effectively controlled hypercalcemia in our population of kidney transplant recipients with hypercalcemic hyperparathyroidism (Figure 2(a)). The mean difference between pre- and posttreatment medians of total serum calcium was −0.30 mmol/L (−0.34 to −0.26 mmol/L, *P* < 0.001, *n* = 41). The decrease in total serum calcium levels was accompanied by an increase in urinary fractional calcium excretion (0.24 (0.06 to 0.42)%, *P* < 0.05, *n* = 40) (Figure 2(b)). Importantly, urolithiasis did not occur in any of the participants. Moreover, serum levels of inorganic phosphate and TmP/GFR simultaneously increased during the first year after treatment start, then reaching a plateau (Figures 3(a) and 3(b)). The mean difference between pre- and posttreatment medians was 0.19 mmol/L (0.15 to 0.23 mmol/L, *P* < 0.001, *n* = 41) for serum levels of inorganic phosphate and 0.20 mmol/L (0.16 to 0.23 mmol/L, *P* < 0.001, *n* = 40) for TmP/GFR. Variations in intact PTH levels over time are depicted in Figure 4. The mean difference between pre- and posttreatment median values of intact PTH was −79 pg/mL (−103 to −55 pg/mL, *P* < 0.001, *n* = 41). Four patients continuously received active vitamin D analogues in addition to cinacalcet starting within three months after initiation of cinacalcet. Another four patients temporarily received active vitamin D analogues in addition to cinacalcet later in the course. After excluding these values from the analysis of intact PTH, the mean difference between pre- and posttreatment medians was smaller, but still significant (−66 (−91 to −41) pg/mL, *P* < 0.001, *n* = 37). None of the participants underwent parathyroidectomy during the entire observation period.

**Figure 5:**
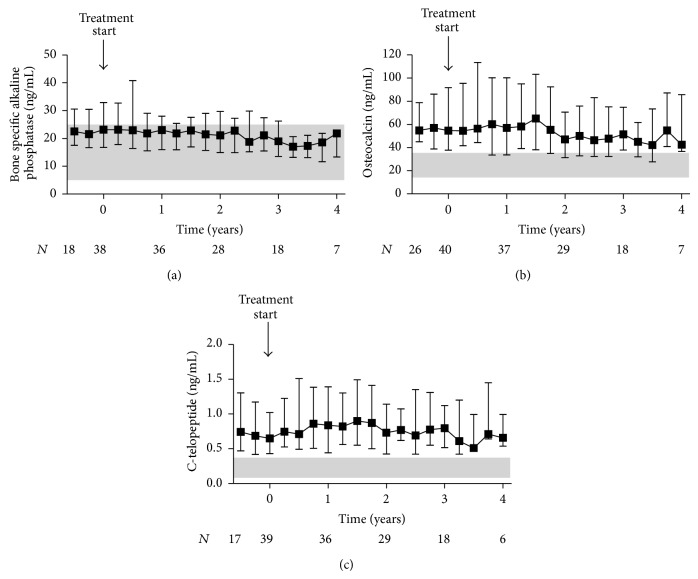
Markers of bone formation and resorption in kidney transplant recipients treated with cinacalcet for hypercalcemic hyperparathyroidism. Variations of (a) bone specific alkaline phosphatase, (b) osteocalcin, and (c) C-telopeptide before and after initiation of cinacalcet (*t* = 0) are plotted over time. Starting point of the pretreatment period is the date when the patients initially presented with hypercalcemia following transplantation. For both pre- and posttreatment period, quarterly median values were obtained for each patient by summarizing all repeated measurements over a three-month period starting from *t* = 0. Data of all patients are presented as medians and interquartile ranges. The shaded area depicts the reference range.

Variations in markers of bone resorption and formation are depicted in Figure 5. C-telopeptide levels remained stable; osteocalcin and bone specific alkaline phosphatase levels decreased over time (*P* < 0.05). The mean difference between pre- and posttreatment median values was 12.5 (1.7 to 23.2) ng/mL for osteocalcin and 5.5 (1.2 to 9.7) ng/mL for bone specific alkaline phosphatase.

Due to the heterogeneity of the study population, we performed a subgroup analysis and compared the effects of cinacalcet between short-term and long-term kidney transplant recipients, using a cut-off value of two years. In general, the effects of cinacalcet on biochemical parameters of mineral metabolism were less pronounced in long-term kidney transplant recipients (i.e., a median of 4.8 (2.5 to 6.0) years after transplantation) as compared with short-term kidney transplant recipients (i.e., a median of 0.8 (0.5 to 0.9) years after transplantation), however, without reaching statistical significance (Table 2).

### 3.3. Discontinuation of Cinacalcet

**Figure 6:**
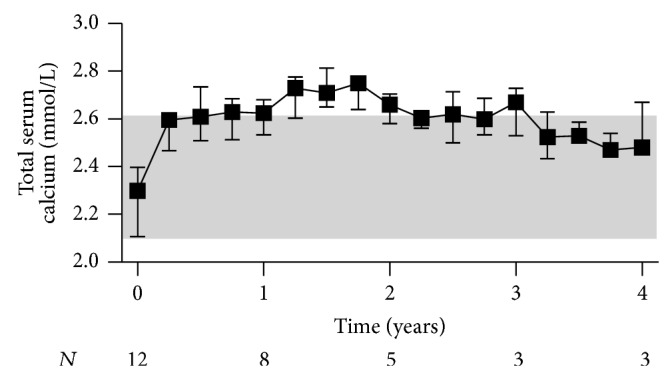
Variations of total serum calcium levels in twelve kidney transplant recipients after discontinuation of cinacalcet. In twelve patients cinacalcet was discontinued and in all of them total serum calcium levels increased after cessation. Cinacalcet was restarted in four patients after a median (IQR) period of 1 (0.7 to 1.5) year. Data are presented as medians and interquartile ranges. The shaded area depicts the reference range.

At some point during the study, cinacalcet was discontinued in twenty patients after a median treatment period of 3.5 (1.6 to 5.0) years. In two patients each cinacalcet was discontinued permanently due to poor tolerance (see safety aspects) and incompliance and because the costs for the medication were not reimbursed by the patients' health insurances. Another two patients declined to continue taking the drug. In twelve patients cinacalcet was discontinued as hypercalcemia was thought to be resolved, with median total serum calcium levels of 2.30 (2.14 to 2.39) mmol/L at the time of drug discontinuation. However, in all of the patients total serum calcium levels increased after cinacalcet cessation (Figure 6) and cinacalcet was restarted in 4 patients after a median period of 1 (0.7 to 1.5) year. Two of these patients eventually returned to dialysis and in six patients treatment is still discontinued, with median total serum calcium levels of 2.57 (2.54 to 2.62) mmol/L reported at the last visit.

### 3.4. Safety Aspects

In total, 19 patients reported side effects following treatment with cinacalcet, according to an overall exposure-adjusted rate of 9.2 per 100 patient-years (5.1 to 13.3). Fourteen patients reported side effects following intake of 30 mg of cinacalcet, four patients following intake of 60 mg of cinacalcet, and one patient following intake of 90 mg of cinacalcet. The majority (79%) had minor gastrointestinal symptoms. A detailed summary of all reported symptoms is given in Table 3. Importantly, in all cases, symptoms were transient and disappeared after dose reduction or switch to twice daily administration. In general, in all participants who needed higher doses than 30 mg, cinacalcet was administered twice daily because of better tolerability. Permanent discontinuation of cinacalcet because of side effects was required in one patient only who showed reversible serious neurological side effects following intake of 30 mg of cinacalcet as reported previously [20]. Moreover, in a patient with HIV, cinacalcet was withdrawn permanently as an interaction between cinacalcet and the antiretroviral medication was suspected because of highly variable levels of tacrolimus.

**Figure 7:**
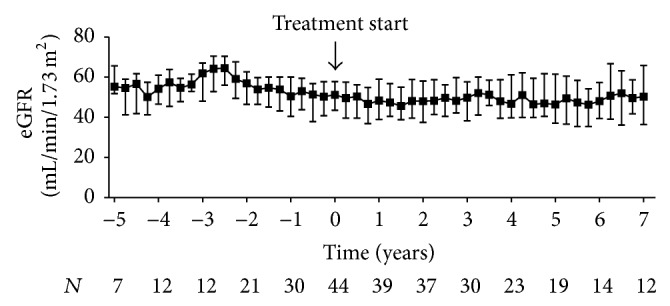
Renal allograft function in kidney transplant recipients treated with cinacalcet for hypercalcemic hyperparathyroidism. Variations of the estimated glomerular filtration rate (eGFR) calculated from serum creatinine using the Modification of Diet in Renal Disease Study equation before and after initiation of cinacalcet (*t* = 0) are plotted over time. Starting point of the pretreatment period is the date when the patients initially presented with hypercalcemia following transplantation. For both pre- and posttreatment period, quarterly median values were obtained for each patient by summarizing all repeated measurements over a three-month period starting from *t* = 0. Data are presented as medians and interquartile ranges.

We did not observe deterioration of allograft function following treatment with cinacalcet, not even in patients who required higher doses. One patient was treated with 180 mg, two patients with 120 mg, and one patient with 90 mg of cinacalcet for five to seven years. Nevertheless, graft function remained stable in these patients (median eGFR at baseline versus last visit: 41.6 (33.7 to 51.2) mL/min/1.73 m^2^ versus 40.5 (30.4 to 52.9) mL/min/1.73 m^2^). Variations in eGFR over time are shown in Figure 7.

## 4. Discussion

In the present study we demonstrated that in kidney transplant recipients with persistent hypercalcemic hyperparathyroidism cinacalcet not only controlled hypercalcemia in the long-term (median treatment period of 6.2 years) but also improved low serum levels of inorganic phosphate without causing hyperphosphatemia. Cinacalcet was well tolerated even after long-term treatment. Thus, this long-term report confirms and extends our knowledge of the efficacy and tolerability of cinacalcet in kidney transplant patients with hypercalcemic hyperparathyroidism obtained by several short-term studies. In addition, a novel indication for cinacalcet in the treatment of posttransplant hypophosphatemia arises from the present study.

Hypercalcemia associated with hyperparathyroidism is a common complication after kidney transplantation [1]. Typically, serum calcium levels decrease in the immediate posttransplant period and then progressively increase and stabilize at approximately six months after transplantation with only minor variations thereafter [22]. In 5% to 25% of cases, serum calcium levels remain elevated even in long-term kidney transplant recipients [3, 23–26]. In accordance with these reports, a cross-sectional analysis at our nephrology outpatient unit revealed that among 288 long-term kidney transplant recipients with a mean eGFR of 38 ± 14 mL/min/1.73 m^2^ at 10 ± 5 years after transplantation, 10% were hypercalcemic and had parathyroid hormone levels above the target range as recommended by The Kidney Disease: Improving Global Outcomes (KDIGO) Clinical Practice Guidelines (Steindl G. and Borchhardt K., unpublished data, 2006).

Another common complication after kidney transplantation is the so-called phosphate leak, which develops as a consequence of persistently high levels of the phosphaturic hormones PTH and fibroblast growth factor 23 (FGF23), and is considered to be a major contributing factor to posttransplant bone disease [7]. Hypophosphatemia occurs mainly during the early posttransplant period, but it may also persist beyond the first year after transplantation to a minor degree [22, 27]. In the present study, 24% of the patients who had received a kidney transplant within less than a year had serum levels of inorganic phosphate below 0.7 mmol/L at baseline, whereas 19% of the patients who had undergone renal transplantation more than one year before did so. The only therapeutic option of normalizing low serum levels of inorganic phosphate is by supplementing inorganic phosphate. However, phosphate supplements should be used with caution in hypercalcemic patients as phosphate loading may lead to the formation of calcium and phosphate precipitates and the development of soft tissue calcifications including renal calcifications and subsequent kidney failure [9–11].

As for the phosphate leak, therapeutic options are limited for persistent hypercalcemia associated with posttransplant hyperparathyroidism. KDIGO Clinical Practice Guidelines for the Care of Kidney Transplant Recipients recommend treating disturbances of calcium, phosphate, and PTH as in nontransplant patients with chronic kidney disease [8], which are, however, not applicable for several reasons. Firstly, the use of active vitamin D analogues in transplant patients with persistent hypercalcemic hyperparathyroidism is limited by hypercalcemia. Secondly, as stated above, kidney transplant patients tend to have low levels of inorganic phosphate in contrast to nontransplanted chronic kidney disease patients, in whom serum levels of inorganic phosphate are usually high, thus requiring the use of phosphate binders. Thirdly, while in patients with chronic kidney disease parathyroidectomy is indicated in case of severe hyperparathyroidism associated with hypercalcemia and/or hyperphosphatemia that cannot be efficiently controlled by medical therapy [28], there are no recommendations available for transplant patients with persistent hypercalcemic hyperparathyroidism. Depending on the institution, some experts prefer early surgical intervention [29], while others recommend waiting for approximately one year for spontaneous resolution of mild to moderate hypercalcemia before therapeutic intervention [30]. One concern about parathyroidectomy in kidney transplant recipients is related to the fact that PTH levels remain below the target range as recommended for chronic kidney disease patients in more than half of the cases [31]. It is expected that persistent hypoparathyroidism after parathyroidectomy might exacerbate low turnover bone disease, which was commonly observed after kidney transplantation [32–34]. Especially in kidney transplant recipients treated with steroids, PTH is considered to play an important role in maintaining bone metabolism [35]. Consequences of persistent and irreversible hypoparathyroidism after parathyroidectomy could be particularly detrimental in case of graft loss and return to dialysis. Hyperparathyroidism is regarded an important adaptive response to decreased renal function in order to maintain mineral and bone metabolism, a regulatory mechanism that is lost after parathyroidectomy.

A more flexible and reversible control of hyperparathyroidism can be achieved by medical treatment with calcimimetics. Calcimimetics increase the sensitivity of the calcium sensing receptor towards calcium, thereby exerting PTH-suppressive and calcium lowering effects [36]. Since cinacalcet has become available for treatment of secondary hyperparathyroidism in chronic dialysis patients [12, 13], there has also been increasing interest in the use of this drug after transplantation (reviewed in [15]). Cinacalcet is not yet approved for the use of hypercalcemic hyperparathyroidism after kidney transplantation but approval is expected in the near future (clinicaltrials.gov NCT00975000). Especially for patients who do not qualify for parathyroidectomy because of only mild or moderate hypercalcemic hyperparathyroidism or in patients who refuse to undergo surgery (both applicable in our study population), cinacalcet offers an alternative therapeutic option. In addition, patients with hypercalcemic hyperparathyroidism and concomitant hypophosphatemia may further benefit from the phosphatemic effect of cinacalcet (reviewed in [15]).

In the present study, serum levels of inorganic phosphate increased by approximately 26% mirroring the increase of 7% to 50% that was reported by previous short-term studies (reviewed in [15]). Simultaneous increases in serum levels of inorganic phosphate and TmP/GFR after treatment start suggest that the correction of hypophosphatemia is caused by a positive balance of phosphate, likely mediated by a reduction in PTH-induced phosphaturia [37]. Whether reduction in the phosphaturic hormone FGF23 also plays a role in the regulation of renal phosphate handling in the long-term remains to be elucidated [38–40]. Notably, hyperphosphatemia did not occur and median serum levels of inorganic phosphate as well as TmP/GFR remained stable with narrow interquartile ranges, possibly reflecting the ability of cinacalcet to establish a steady state in the long-term. This is of particular importance as high serum levels of inorganic phosphate were identified as an independent predictor of all-cause mortality in patients after kidney transplantation, with patients with serum levels of inorganic phosphate above only 1.12 mmol/L having the greatest increase in mortality [41].

Regarding calcium homeostasis, we achieved a reduction in total serum calcium levels by 11%, which is in accordance with previous short-term studies that reported a decrease of 7% to 15% (reviewed in [15]). Even though some participants had suffered from hypercalcemic hyperparathyroidism over years, they responded to cinacalcet, only four of them required higher doses. Kruse and colleagues described beneficial effects of cinacalcet on serum calcium levels even after drug withdrawal after twelve months of treatment, with hypercalcemia being persistently controlled in half of the patients at nine months after cessation [42]. In the present study, even after a prolonged treatment period, all of the patients in whom a good control of hypercalcemia was achieved beforehand experienced recurrence of hypercalcemia after cessation of cinacalcet.

As demonstrated by pharmacokinetic studies, the calcium lowering effect of cinacalcet is considered to be mediated by increased urinary calcium excretion [43, 44]. Cinacalcet likely increases urinary calcium excretion indirectly by lowering PTH levels as well as by targeting the renal calcium sensing receptor, thus directly modulating renal calcium handling [45]. Data on whether this increase is sustained over time are controversial. While some studies did not find a difference in urinary calcium excretion following treatment with cinacalcet up to twelve months [43, 46, 47], others, including our own, reported a sustained increase [20, 48]. Importantly, Courbebaisse and colleagues observed no difference in calcification in kidney graft biopsies after twelve months of treatment with cinacalcet in comparison to patients not treated with cinacalcet [48]. In a recent case report, calciuria and renal calcifications occurred following treatment with a high dose of cinacalcet (180 mg), however, without any deterioration of allograft function [49]. In contrast, in two other cases, development of nephrocalcinosis and acute graft failure was reported after cinacalcet was given to kidney transplant recipients with severe forms of hyperparathyroidism at 30 mg [50, 51]. The authors hypothesized that treatment with cinacalcet in the immediate posttransplant period, when calcineurin inhibitors and high doses of glucocorticoids enhance bone resorption and calcium excretion, might have contributed to acute renal failure [51]. In the present study, four patients received high doses of cinacalcet between five and seven years. Nevertheless, graft function remained stable in these patients.

In general, data on the effect of cinacalcet on graft function are contradictory. While some studies reported a stable graft function following treatment with cinacalcet after short-term use [17, 20, 46, 52–56], others observed a deterioration of renal allograft function [57–60]. The population reported by El-Amm et al. had experienced a decline in graft function already before the initiation of cinacalcet which continued but did not further deteriorate after treatment start [57]. The patients investigated by Kruse et al. showed slightly higher serum creatinine levels at baseline [58] which might have made them more susceptible to deterioration of graft function following cinacalcet therapy. In a follow-up study Kruse et al. demonstrated that renal function restores after withdrawal of cinacalcet, suggesting hemodynamic effects of calcium or PTH rather than structural changes [42]. This is supported by the observation of renal function deteriorations within the first weeks after parathyroidectomy without affecting the long-term graft outcome [61].

So far, only a few small studies investigated how cinacalcet influences bone metabolism, structure, and density. Only one retrospective case control study reported a slight increase in bone mineral density in femoral neck at three years after transplantation following treatment with cinacalcet between one and two years [62]. Moreover, cinacalcet was shown to reduce bone formation rate in both hemodialysis patients [63, 64] and kidney transplant recipients with hyperparathyroidism as we have previously demonstrated [65]. In the present study we assessed serum markers of bone formation and resorption, which likely confirms reduced bone turnover over time. Due to the limited number of measurements, however, these data need to be interpreted with caution. Notably, during the long-term follow-up, only one participant sustained a rib fracture, which was discovered incidentally one year after transplantation on a chest X-ray. By the time the fracture was discovered the patient had received cinacalcet at an average daily dose of 25 mg for approximately eleven months. Cinacalcet was then paused for two months and restarted at a daily dose of 15 mg as total serum calcium levels had again increased. As this fracture occurred during the early posttransplant period, we consider it most likely related to steroid-induced bone loss and the associated increase in fracture risk [66]. No other fractures occurred during the study. Further data on the effect of cinacalcet on bone density and fractures in kidney transplant recipients are expected from a currently ongoing randomized controlled trial (NCT00975000), which, however, has a follow-up of only one year. We believe that in future, the performance of long-term studies like this will be challenging especially because cinacalcet is widely used in dialysis patients and frequently continued at lower doses after transplantation. We are convinced that, despite its limitations, the present study provides valuable experience on the long-term use of cinacalcet after transplantation, which is certainly not feasible to be reproduced in a randomized controlled setting.

## 5. Conclusions

In conclusion, results from our long-term study in kidney transplant patients indicate that cinacalcet not only controls hypercalcemic hyperparathyroidism but also corrects low serum levels of inorganic phosphate without causing hyperphosphatemia. Thus, it offers an ideal treatment for hypercalcemic kidney transplant recipients with concomitant hypophosphatemia, which is otherwise difficult to treat due to the risk of soft tissue calcification possibly associated with phosphate supplementation. Overall, cinacalcet was well tolerated and side effects could be minimized by twice daily administration.

## Figures and Tables

**Table 1:** Baseline characteristics of 44 patients treated with cinacalcet for hypercalcemic hyperparathyroidism following kidney transplantation (KT).

	Median (IQR)/count (frequency, %)
Age, years	56 (48–61)
Men, *N* (%)	29 (66)
Deceased-donor KT, *N* (%)	41 (93)
Previous transplants: 0/1/2, *N* (%)	36/4/4 (82/9/9)
Dialysis prior to KT, *N* (%)	42 (96)
Time on dialysis prior to KT, years	3.2 (2.4–4.3)
Time between first renal replacement therapy until inclusion, years	6.0 (4.5–10.3)
Time between last KT and development of hypercalcemia, months	1.0 (0.5–2.1)
Time between last KT and cinacalcet initiation, years	1.8 (0.8–4.7)
Laboratory findings, unit (reference range)	
Estimated glomerular filtration rate, mL/min/1.73 m^2^	51.2 (43.5–57.8)
Total serum calcium, mmol/L (2.10–2.60)	2.77 (2.68–2.84)
Fractional calcium excretion, % (1-2)	0.77 (0.44–1.45)
Serum inorganic phosphate, mmol/L (0.81–1.45)	0.79 (0.72–0.89)
TmP/GFR, mmol/L (0.80–1.40)	0.54 (0.46–0.63)
Intact parathyroid hormone, pg/mL	156 (112–236)
Bone specific alkaline phosphatase, ng/mL (5.2–24.6)	23.1 (16.8–32.9)
Osteocalcin, ng/mL (15–35)	54.6 (37.7–91.8)
C-telopeptide, ng/mL (0.08–0.44)	0.65 (0.43–1.02)
Native kidney disease, *N* (%)	
Polycystic disease	12 27.3
Glomerular disease	11 25.0
Vascular disease	3 6.8
Diabetes mellitus	2 4.5
Others	7 15.9
Unknown	9 20.5
Immunosuppressive therapy, *N* (%)	
Cyclosporine A/tacrolimus	17/22 (38.6/50.0)
Mycophenolate mofetil/azathioprine/leflunomide	40/1/1 (90.9/2.3/2.3)
Sirolimus/everolimus	1/1 (2.3/2.3)
Belatacept	2 4.5
Steroids	40 90.9
Pretransplant use of cinacalcet, *N* (%)	13 29.5

IQR, interquartile range; KT, kidney transplantation; TmP/GFR, renal tubular reabsorption of phosphate to glomerular filtration rate.

**Table 2:** Effect of cinacalcet on biochemical parameters of mineral metabolism in short- and long-term kidney transplant recipients.

	Total cohort *n* = 44	Short-term (≤2 years)	Long-term (>2 years)
		Kidney transplant recipients	
		*n* = 19	*n* = 22
Total serum calcium, mmol/L	−0.30 (−0.34 to −0.26)	−0.33 (−0.38 to −0.27)	−0.26 (−0.32 to −0.21)
Fractional calcium excretion, %	0.24 (0.06 to 0.42)	0.28 (0.01 to 0.55)	0.19 (−0.06 to 0.44)
Serum inorganic phosphate, mmol/L	0.19 (0.15 to 0.23)	0.21 (0.14 to 0.27)	0.17 (0.13 to 0.21)
TmP/GFR, mmol/L	0.20 (0.16 to 0.23)	0.21 (0.15 to 0.26)	0.18 (0.13 to 0.23)
Intact parathyroid hormone, pg/mL	−66 (−91 to −41)	−84 (−128 to −40)	−45 (−64 to −26)

TmP/GFR, renal tubular reabsorption of phosphate to glomerular filtration rate; mean differences (95% confidence intervals) between pre- and posttreatment median values of biochemical parameters are presented for short-term (i.e., a median of 0.8 (0.5 to 0.9) years posttransplant) and long-term (i.e., a median of 4.8 (2.5 to 6.8) years posttransplant) kidney transplant recipients.

**Table 3:** Side effects reported by 19 out of 44 kidney transplant recipients following treatment with cinacalcet.

Symptoms	*N*
Gastrointestinal symptoms	
Nausea	7
Abdominal cramps	5
Vomiting	3
Diarrhea	2
Epigastralgia	2
Neurological symptoms	
Paresthesia	3
Vertigo	1
Nervousness	1
Deterioration of hearing loss	1
Serious neurological symptoms [20]	1
Other symptoms	
Myalgia	3
Attacks of sweating	1
Pruritus	1
